# Alteration in Amino Acid Metabolism After Isocaloric, Energy-Restricted Ketogenic Diet in Women with Overweight and Obesity: Randomized KETO-MINOX Trial

**DOI:** 10.3390/nu18020300

**Published:** 2026-01-18

**Authors:** Natalia Drabińska-Fois, Anna Majcher, Paweł Jagielski, Sebastian Borowicz-Skoneczny, Jerzy Romaszko

**Affiliations:** 1 Food Volatilomics and Sensomics Group, Department of Food Technology of Plant Origin, Faculty of Food Science and Nutrition, Poznan University of Life Sciences, Wojska Polskiego 28, 60-637 Poznań, Poland; 2 Institute of Animal Reproduction and Food Research, Polish Academy of Sciences, Tuwima 10 Str., 10-748 Olsztyn, Poland; majcher2003ania@gmail.com; 3 Department of Nutrition and Drug Research, Institute of Public Health, Faculty of Health Sciences, Jagiellonian University Medical College, 31-066 Krakow, Poland; paweljan.jagielski@uj.edu.pl; 4 Department of Family Medicine and Infectious Diseases, University of Warmia and Mazury in Olsztyn, 10-082 Olsztyn, Poland; morowo1994@gmail.com (S.B.-S.); jerzy.romaszko@uwm.edu.pl (J.R.)

**Keywords:** ketogenic diet, overweight, obesity, amino acids, metabolism

## Abstract

**Background/Objectives**: Circulating amino acid concentrations and their excretion can provide insights into dietary protein intake and metabolism. Alterations in amino acid homeostasis occur in various disorders due to nutritional imbalances or metabolic changes, including obesity. A ketogenic diet (KD) has gained popularity for weight management; however, its metabolic effects are not fully known. Therefore, the aim of this study was to evaluate the effect of an eight-week, energy-restricted Mediterranean-type KD on the amino acid metabolism in women with overweight and class I obesity. **Methods**: A randomized, single-center, controlled trial was conducted with 80 women with a BMI of 25.5–35 in age between 18 and 45 years, without any chronic diseases. Randomly divided women received food catering with approximately 1750 kcal daily for eight weeks, containing KD or standard diet (STD), respectively. The concentration of amino acids was assessed by gas chromatography-mass spectrometry after the derivatization with chloroformate in serum and urine collected at the baseline, after 4 weeks, and at the end of the intervention. **Results**: The results collected from 66 participants were included in the final analyses. Independent of diet type, weight reduction was associated with increased circulating α-aminobutyric acid and decreased proline, glutamate, and tyrosine. The KD led to lower concentrations of alanine, methionine, threonine, and tryptophan, alongside higher levels of branched-chain amino acids (BCAA) and α-aminobutyric acid compared to the STD. Urinary amino acid excretion decreased after weight reduction. KD was associated with higher urinary excretion of BCAA and β-aminoisobutyric acid. **Conclusions**: In summary, both weight reduction and KD significantly affect the amino acid metabolism, which might have implications for inflammation, oxidative stress, and cardiometabolic risk.

## 1. Introduction

Obesity is a highly prevalent, chronic, and multifactorial disease associated with numerous adverse health outcomes, ultimately contributing to reduced life expectancy [1]. Currently, body mass index (BMI), calculated from weight and height, is the standard metric for assessing obesity severity: BMI 25–30 is classified as overweight, BMI 30–35 as class I obesity, BMI 35–40 as class II obesity, and BMI > 40 as class III obesity [2]. However, BMI has well-recognized limitations, which raise the discussion about its utility. BMI does not accurately reflect visceral adiposity, which is strongly linked to the development of metabolic comorbidities [3]. That is why BMI and other anthropometric measures have to be used with caution.

Treatment strategies vary according to the severity of obesity. For severe obesity, pharmacotherapy and bariatric surgery are commonly employed, whereas lifestyle interventions such as increased physical activity, energy-restricted diets, and consumption of satiety-promoting foods are typically recommended for individuals with overweight and class I obesity [3,4,5]. Importantly, recent evidence highlights sex-specific differences in obesity prevalence and pathophysiology, underscoring the need for tailored weight-loss interventions [6].

Amino acids, organic compounds containing carboxyl and amine groups, play essential roles in human metabolism and physiology [7]. Circulating amino acid concentrations are influenced by multiple factors, including protein catabolism, extracellular release, protein synthesis, tissue-specific uptake, oxidative metabolism, and excretion of amino acids and their catabolic products [8]. In obesity, amino acid profiles are markedly altered, with elevated levels of branched-chain amino acids (BCAAs; leucine, isoleucine, valine) and aromatic amino acids (phenylalanine, tyrosine) [9,10]. Increased BCAA and methionine concentrations have been proposed as early indicators of insulin resistance [8]. Mechanistically, elevated free fatty acids in obesity may lead to mitochondrial acetyl-CoA accumulation, disrupting redox balance and inhibiting key enzymes such as branched-chain ketoacid dehydrogenase (BCKD) and pyruvate dehydrogenase (PDH), thereby impairing BCAA and pyruvate metabolism. Additionally, oxidative stress promotes methionine catabolism into glutathione, generating cysteine and cystine, which inhibit tyrosine aminotransferase and slow tyrosine degradation [8]. Notably, amino acid concentrations also change during weight-loss interventions [11]. It was shown that the concentration of alanine, isoleucine, tyrosine, phenylalanine, and glutamic acid decreased after weight reduction, whereas asparagine increases during weight maintenance.

The ketogenic diet (KD), characterized by high fat and low carbohydrate intake, which leads to a ketosis state, has gained popularity as a weight-loss strategy [12]. KD induces a metabolic shift from glucose utilization to ketone body oxidation, mimicking the physiological state of fasting [13]. Despite its widespread popularity, the long-term metabolic consequences of KD remain incompletely understood. Many existing studies lack control groups, employ extreme caloric restriction (500–700 kcal/day), involve small sample sizes, or are limited to short intervention periods [14]. An important element of KD is adaptation to ketosis, which typically requires 4–6 weeks, during which temporary side effects, commonly referred to as “ketogenic flu” may occur, including headache, nausea, and fatigue. Therefore, extended adherence is necessary to evaluate the true metabolic impact of KD.

Metabolomic studies investigating KD have primarily focused on patients with epilepsy, for whom KD was originally developed [15,16]. These studies report that KD led to increased circulating gamma-aminobutyric acid (GABA) and its precursor glutamine [16], as well as alterations in cerebrospinal fluid amino acid profiles, including elevated taurine, serine, and glycine and reduced alanine, tyrosine, and phenylalanine [17]. Notably, the authors also reported a significant correlation between the level of threonine and seizure response [17]. In healthy adults, short-term KD (three weeks, thus the adaptation to ketosis has not been achieved) increased BCAA concentrations while reducing glucogenic amino acids such as alanine, glutamine, and proline [18]. To date, no studies have examined the impact of KD on amino acid metabolism in individuals with overweight or obesity, particularly women, despite the distinct metabolic and hormonal differences observed in female cohorts.

This gap in knowledge forms the basis of the present study, which aims to evaluate the effects of an energy-restricted KD on serum and urinary amino acid profiles in women with overweight and obesity. By profiling both serum and urine, and by employing an energy-restricted Mediterranean-type KD sustained beyond the ketosis-adaptation window, the study aims to deliver translational insights that can improve diet selection, monitoring, and personalization in clinical weight-management programs.

## 2. Materials and Methods

### 2.1. Study Design

A randomized, single-center, clinical trial with a nutritional intervention was performed in Olsztyn (Poland). The detailed description of the study design and eligibility criteria can be found in the previously published study protocol [19] and in the U.S. National Library of Medicine (ID: NCT05652972; http://www.clinicaltrials.gov, accessed on 10 May 2023)). The study was approved by the Bioethics Committee of the Faculty of Medical Sciences of the University of Warmia and Mazury in Olsztyn (agreement No: 25/2022 from 27 October 2022).

Eighty women with overweight and class I obesity (BMI: 25.5–35.0 km/m^2^) were enrolled in the study and randomly divided into two groups: KETO—the group following KD; or the STD—the control group receiving a standard diet. Both groups received the corresponding diet in the form of four-meal catering of approximately 1750 kcal, which was delivered every day to each participant for the whole period of nutritional intervention by BE KETO Catering company (https://beketocatering.pl, accessed on 20 December 2025). All participants within each group received identical meals each day. KETO and STD groups received meals with a macronutrient composition of 70:20:10 and 20:30:50 fat: protein: carbohydrate ratio, respectively. Irrespectively of the macronutrient distribution, both diets were considered healthy and complied with the principles of the Mediterranean-type diet. The check-up visits and sample collection were conducted at the baseline (T0), after four weeks (T1), and at the end of the intervention, after 8 weeks (T2). During each visit, the body weight and composition were measured using a TANITA Body Composition Analyzer (Tanita Polska, Poznań, Poland), while the waist and hip circumferences were measured using a standard measurement tape (Tanita Polska, Poznań, Poland). The blood samples were collected by a qualified nurse after overnight fasting into a vacuum tube for serum analyses. The tubes were centrifuged at 3500 rpm for 10 min, and the serum samples were aliquoted and stored until analysis in an ultra-freezer at −80 °C. Morning urine samples were delivered by participants for the check-up visits in the sterile urine collection containers, aliquoted, and stored until analyses in an ultra-freezer at −80 °C.

### 2.2. Amino Acid Analysis

The free amino acids in urine and serum were analyzed using a derivatization with propyl chloroformate. Briefly, 200 µL of urine or plasma was mixed with 200 µL of 1 M NaOH, 80 µL of internal standard (norvaline), and 80 µL of pyridine in 1-propanol (1:3, *v*:*v*) and mixed in a vortex for 1 min. Then 200 µL of propyl chloroformate in chloroform (1:9, *v*:*v*) was added, and the mixture was vortexed for 1 min. Next, the mixtures were allowed to stand for 2.5 min. The vials were centrifuged for 1 min at 13,500 rpm, and the bottom organic layer was transferred into a glass insert in a 2 mL GC vial.

Amino acids were analyzed using an Agilent 7890A gas chromatograph (Agilent Technologies, Santa Clara, CA, USA) coupled to a 5975C mass spectrometer (Agilent Technologies, Santa Clara, CA, USA). Individual compounds were separated in the ZBAAA EZ Faast™ capillary column (10 m × 0.25 mm). The carrier gas was helium (1.5 mL/min). The samples (1 μL) were injected in split mode (1:10). Oven temperature was initially set at 110 °C and then increased to 320 °C with 30 °C/min. Injector and MS source temperatures were set at 250 and 240 °C, respectively. Mass spectra were obtained by electron ionization (EI) over the range of 35–550 m/e. Total ion chromatograms were analyzed using MassHunter software version 10.0 (Agilent Technologies, Santa Clara, CA, USA). Individual amino acids were identified using external standards for each AA, and normalized in relation to the internal standard.

The concentration of free amino acids in urine was normalized in relation to the specific gravity, measured by the Diagnostic Laboratory.

### 2.3. Statistical Analysis

Only data from participants who adhered to the study protocol for at least 80% of the intervention period were included in the final analysis. The normality of the distribution of the analyzed quantitative variables was tested using the Shapiro–Wilk W test. Results for quantitative variables with a non-normal distribution are presented as the median and the lower (Q25) and upper (Q75) quartiles. The Mann–Whitney U test was used to test for differences in variables depending on diet type, and the Wilcoxon test was used to test for statistically significant changes over time. For the three time points, the Friedman test was used to test for differences over time. The statistical analyses were performed using PS IMAGO PRO 10 software (IBM SPSS Statistics 29). The level of statistical significance was set at α = 0.05.

## 3. Results

**Figure 1:**
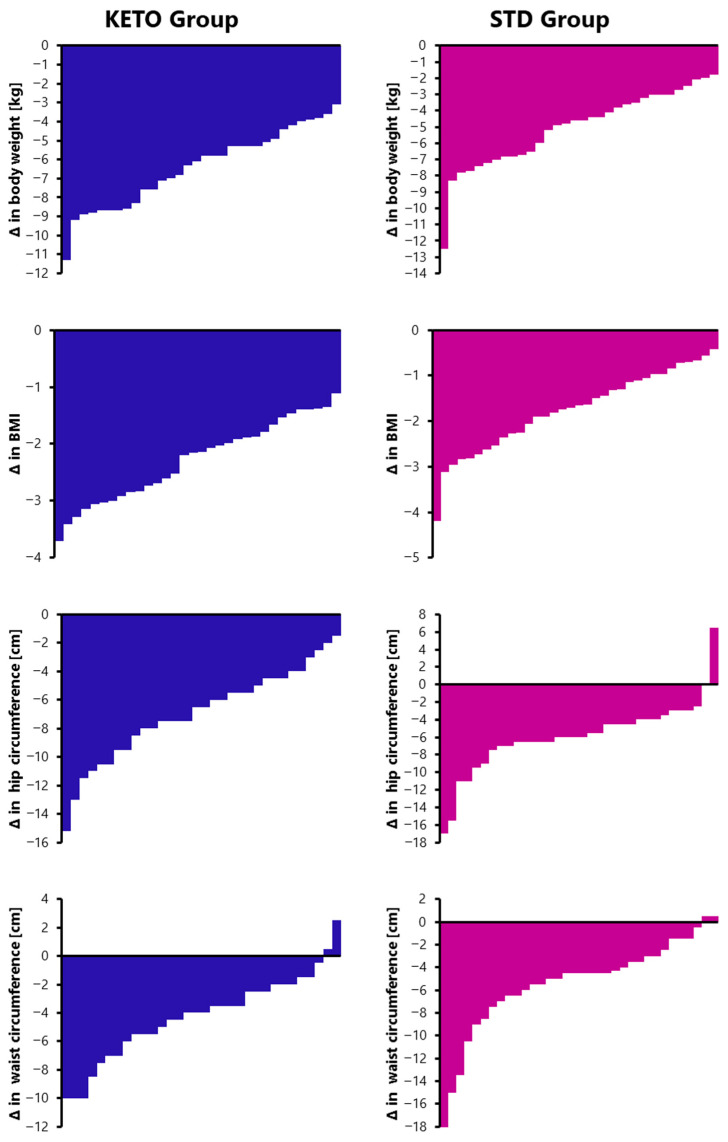
The changes in the body weight, BMI, hip, and waist circumferences between T0 and T2 in the individuals from KETO (blue) and STD (purple) groups.

The changes in anthropometric indices for each participant in the KETO and STD groups are presented in Figure 1. Regardless of diet composition, both groups exhibited significant reductions in body weight, BMI, and waist and hip circumferences. Participants in the KETO group lost significantly more body weight than those in the STD group (mean change: −6.42 ± 2.03 kg vs. −4.85 ± 2.44 kg; *p* = 0.014), and consequently showed a greater reduction in BMI (−2.29 ± 0.71 vs. −1.76 ± 0.88; *p* = 0.022). No significant differences were observed for waist (−4.27 ± 2.29 vs. −5.42 ± 4.13) or hip circumferences (−6.94 ± 3.23 vs. −5.90 ± 4.07) between the study groups.

Interestingly, although body weight decreased in all participants, one individual in the STD group exhibited an increase in hip circumference, and two participants from each study group showed an increase in waist circumference.

### 3.1. Concentration of Free Amino Acids in Serum

The concentrations of individual amino acids in serum samples are presented in Table 1. In serum samples, in total 19 free amino acids were identified, including eight non-essential, eight essential, and three other amino acids. At baseline, the concentrations of most free amino acids did not differ between the KETO and STD groups, except for aspartic acid.

In the KETO group, a significant reduction was observed in the concentration of alanine, proline, methionine, phenylalanine, aspartic acid, glutamic acid, and tyrosine. Conversely, the concentration of α-aminobutyric acid, valine, and leucine increased over the course of intervention. The level of serine, ornithine, and lysine decreased after the first four weeks, but subsequently increased to values exceeding baseline concentrations by the end of the intervention. In the STD group, a significant increase was noted in the concentration of α-aminobutyric acid, ornithine, lysine, asparagine, and tryptophan, while decreases were observed in proline, glutamic acid, and tyrosine during the weight reduction.

The comparison between the effects observed in the KETO and STD groups showed significant differences at T1 in the concentration of alanine, α-aminobutyric acid, valine, leucine, isoleucine, threonine, methionine, and tryptophan. At T2, significant differences were observed for alanine, α-aminobutyric acid, valine, leucine, isoleucine, threonine, methionine, ornithine, lysine, and asparagine. When normalized to the baseline values, the significant differences were found at T1 for alanine, α-aminobutyric acid, valine, methionine, and asparagine, while at T2, significant differences persisted for alanine, valine, and asparagine.

### 3.2. Urine Excretion of Amino Acids

The results of urinary excretion of free amino acids at the baseline and at T2 in the KETO and STD groups are presented in Table 2. In total, 20 free amino acids were identified in urine samples, including eight non-essential, nine essential, and three other amino acids. At baseline, no differences were observed in urinary free amino acid concentrations between the KETO and STD groups.

In the KETO group, a significant increase in the urinary excretion of β-aminoisobutyric acid, proline, and phenylalanine was observed following the nutritional intervention. Conversely, decreases were noted for alanine, glycine, threonine, serine, methionine, glutamic acid, α-aminoadipic acid, tyrosine, and cystine. In the STD group, urinary concentrations of proline, phenylalanine, and tryptophan increased at T2, whereas concentrations of leucine, threonine, serine, methionine, glutamic acid, α-aminoadipic acid, lysine, and cystine decreased.

The comparison of the effects between the KETO and STD groups revealed significant differences in urinary excretion of valine, β-aminoisobutyric acid, leucine, and tyrosine.

## 4. Discussion

This randomized, controlled trial investigated the effect of an energy-restricted KD in comparison to STD diet with the same calorie intake on the amino acid metabolism in women with overweight or class I obesity. Both interventions used Mediterranean-style versions of KD and STD, emphasizing monounsaturated and polyunsaturated fats (e.g., extra-virgin olive oil, nuts), high dietary fiber, diverse non-starchy vegetables, and polyphenol-rich foods. The diets were implemented with a moderate caloric deficit to evaluate metabolic effects under nutritionally balanced conditions and to provide structured dietary education.

It was found that irrespective of the diet composition, eight weeks of isocaloric and energy-restricted diets increased the circulating level of α-aminobutyric acid and reduced the concentrations of proline, glutamic acid, and tyrosine. α-Aminobutyric acid is a non-proteinogenic amino acid generated from cysteine, methionine, threonine, serine, or glycine [20]. Prior studies report that α-aminobutyric acid can reduce inflammation by modulating macrophage polarization [20] and support myocardial glutathione homeostasis, potentially protecting against oxidative stress [21]. Proline and glutamic acid are glucogenic amino acids; thus, their reduction is consistent with overall caloric restriction and decreased net proteolysis under both diets. Elevated levels of tyrosine have been previously reported in obesity; thus, its reduction with weight loss in both groups aligns with previous findings [9,10,22].

Interestingly, the concentration of ornithine and lysine increased in the STD group while in the KETO group decreased during the adaptation to ketosis (T1), then was followed by a decrease just like in the STD group. When the body adapts to ketosis, and the intake of carbohydrates is very low, the cells reduce glycolysis and increase reliance on gluconeogenesis and amino acid metabolism. Lysine is a strictly ketogenic amino acid and is catabolized to acetoacetyl-CoA, which shifts in whole-body fuel selection, diet protein sources, and transient changes in amino-acid transport and oxidation could contribute to the initial decrease and later rebound of circulating lysine in the KETO group. Ornithine is a part of the urea cycle, which removes excess nitrogen from amino acid breakdown. During ketosis, protein breakdown and amino acid use for gluconeogenesis rise initially, so the urea cycle is more active, contributing to increased ornithine concentration [23]. However, these time-course effects warrant further mechanistic evaluation.

Between groups, alanine, methionine, and tryptophan were significantly lower in KETO versus STD. Alanine, a principal glucogenic amino acid and key substrate in the glucose-alanine (Cahill) cycle, might be reduced under KD due to lower glycolytic flux in muscle and greater hepatic utilization for gluconeogenesis [24]. This difference persisted after normalization to baseline, which could also reflect differences in protein intake and/or sources between diets. Lower methionine in KD, which was maintained also after normalization to baseline values, might relate to diet composition (methionine intake or source), altered transsulfuration and glutathione metabolism, and/or changes in redox status often reported with KD [13]. It was found that the reactive oxygen species affect methionine metabolism [25,26]. Given that higher methionine levels have been associated with insulin resistance [8], one of the possible explanations for the observed reduction might be consistent with improved insulin sensitivity in KD participants, as reported by others [18,27]. Interestingly, the level of asparagine decreased in the KETO group, while in the STD group a slight, non-significant increase was observed. This may reflect altered glutamine/asparagine interconversion and shifts toward oxidative phosphorylation in the presence of higher α-aminobutyric acid [20], though direct mechanistic confirmation is needed.

Threonine was lower in KD than STD at T1 and T2, while α-aminobutyric acid was higher in KD at both time points. Because threonine is a precursor for α-aminobutyric acid, increased α-aminobutyric acid in KD might, in part, reflect enhanced threonine catabolism [21].

Furthermore, in the KETO group, the significantly higher concentrations of BCAA were noted compared to the STD group, also after normalization to the baseline values, which is in agreement with previous studies showing the increase in BCAA after following KD [15,18]. The differences in BCAA, sulfur-containing, and aromatic amino acids in obesity might be explained by modifications in BCKD and mitochondrial branched-chain aminotransferase (BCATm) [28]. Both enzymes are a part of the mitochondrial matrix metabolon, which works together to obtain energy from BCAA. In liver and white adipose tissue (WAT), BCKD can switch between active and inactive forms easily, so its activity changes a lot with fasting or obesity. The phosphorylation, which turns off the BCKD activity, has been previously reported in obesity [29]. Moreover, in obesity, amino acid concentrations increase because of impaired insulin action and elevated free fatty acids that create a metabolic state similar to fasting. Enhanced fat oxidation in the liver and muscle raises mitochondrial NADH and acetyl-CoA levels, which further inhibit key enzymes like BCKD. This inhibition slows BCAA and sulfur amino acid catabolism, causing their accumulation in tissues and blood. Additionally, the reduced activity of BCKD and related dehydrogenases under these conditions promotes the buildup of keto-acid derivatives such as α-KB and α-HB [8]. Thus, the elevated amino acid levels observed in obesity can be explained by the combination of high fat oxidation and low insulin signaling, which shifts metabolism away from amino acid disposal. KD biochemically mimics aspects of fasting; thus, even with reductions in weight and fat mass over the intervention, KD may simultaneously promote (via energy restriction) and inhibit (via BCKDH regulation) BCAA disposal, yielding the observed net elevation. However, the exact mechanism of these changes requires confirmation in the mechanistic studies.

Urinary excretion of amino acids reflects dietary intake, metabolic state, renal handling, and various disease states [30]; therefore, interpreting amino acid levels in urine is challenging and must be approached with caution In our study, in both study groups an increase in the urinary concentration of proline and phenylalanine, whereas threonine, serine, methionine, glutamic acid, α-aminoadipic acid, lysine, and cystine decreased. These changes likely relate to weight loss, not the composition of the diet. Evidence on weight-loss effects on urinary amino acids remains limited. However, it might be speculated that the overall lower excretion of amino acids after weight reduction is due to improved metabolic efficiency and reduced protein metabolism. Weight loss improves insulin sensitivity, which enhances amino acid uptake into tissues for protein synthesis, and there are consequently fewer amino acids to be removed with urine [31]. Hydration status, acid–base balance, and renal transporter competition can also influence these patterns and should be considered in interpretation.

The significant differences between the study groups in terms of absolute values, as well as normalized to the baseline values, showed significantly higher excretion of β-aminoisobutyric acid, valine, and leucine, and at the same time significant reduction in tyrosine concentration in the KETO group. Higher urinary BCAA likely mirrors higher circulating concentrations and incomplete tissue utilization. Although data on urinary β-aminoisobutyric acid in nutritional KD are sparse, greater excretion could reflect higher systemic production and rapid renal clearance, even when serum levels are below the assay’s detection limit. β-Aminoisobutyric acid has been proposed as a myokine-like metabolite that promotes browning of white adipose tissue and associates inversely with cardiometabolic risk [32,33]. While KD can increase total urinary nitrogen loss [34,35], detailed characterizations of individual urinary amino acids during energy-restricted, Mediterranean-type KD in otherwise healthy adults are scarce.

Although both interventions were designed to be isocaloric and Mediterranean-style, differences in total protein intake, amino acid composition, and protein sources may have contributed to the observed concentrations of circulating and urinary amino acids. Interestingly, despite the lower protein intake in the KETO group, serum total essential amino acid levels decreased to a similar extent in both study groups. The decline observed during the first four weeks may be attributable to the overall lower protein intake during the nutritional intervention compared with the habitual pre-study diets of participants, which had previously resulted in increased body weight. The absence of difference in the total essential amino acid level between the KETO and STD groups might reflect metabolic adaptations associated with the ketosis state, such as reduced amino acid oxidation and/or alterations in protein turnover.

Despite the novel nature of this study, some limitations could not be avoided. The study population was limited only to women, which limits generalizability. However, considering the gender differences in the pathophysiology of obesity, we decided that presenting the effects in a more homogenous cohort would provide more valid results. Moreover, the present study was conducted with Mediterranean-type diets and should be extended also to more realistic, Western-type diets. Secondly, all participants were in general healthy; therefore, the results of this study might not apply to individuals suffering from obesity accompanied by cardiometabolic diseases. Additionally, the sample size, although sufficient according to the sample size calculation, might be insufficient to detect plausible interactions of interventions. Finally, the study was conducted over only eight weeks, which corresponds to the typical duration of a weight-reduction program but was sufficient to allow participants to adapt to ketosis (T1) and to assess the effects of the diet once metabolic conditions had stabilized (T2). Extending the duration of the intervention might have further amplified the metabolic changes observed in our study, including more pronounced alterations in glucose and fatty acid metabolism. A longer intervention period could therefore potentially reveal stronger or more sustained metabolic adaptations than those captured within the current timeframe.

## 5. Conclusions

In this study, the effect of KD on the amino acid metabolism in women with overweight and class I obesity was described. Our study demonstrated that both energy-restricted ketogenic and standard Mediterranean-type diets, when matched for caloric intake, significantly influence amino-acid metabolism in women with overweight or class I obesity. Independent of diet type, weight reduction was associated with increased circulating α-aminobutyric acid and decreased proline, glutamate, and tyrosine, changes that might reflect improved metabolic efficiency and reduced inflammation.

The KD led to lower concentrations of alanine, methionine, threonine, and tryptophan, alongside higher levels of BCAA and α-aminobutyric acid compared to the STD. These findings suggest that KD alters amino acid utilization pathways, favoring ketogenic substrates and modulating redox balance, which may contribute to improved insulin sensitivity. Time-dependent changes in ornithine and lysine during KD adaptation highlight dynamic shifts in nitrogen disposal and urea-cycle activity under carbohydrate restriction. Urinary amino acid excretion decreased after weight reduction, likely due to enhanced insulin sensitivity and reduced protein catabolism. However, KD was associated with higher urinary excretion of BCAA and β-aminoisobutyric acid, indicating incomplete tissue utilization and potential metabolic signaling roles for β-aminoisobutyric acid.

In summary, the observed changes in circulating and urinary amino acids may have implications for inflammation, oxidative stress, and cardiometabolic risk. Further research is needed to confirm these mechanisms, explore sex-specific responses, and evaluate long-term effects in diverse populations and dietary patterns.

## Figures and Tables

**Table 1:** Serum concentrations of free amino acids and derivatives in KETO and STD groups before and at the end of the nutritional intervention. Data are given as the median (Q25; Q75) in nmol/mL.

	KETO Group					STD Group				
	T0	T1	T2	Δ T2–T0	*p*-Value ^1^	T0	T1	T2	Δ T2–T0	*p*-Value ^1^
Non-essential amino acids										
ALA	424.3 (362.4; 485.9)	318.8 (291.1; 368.8)	317.1 (284.2; 362.6)	−112.5 (−170; −21.1)	**0.0004**	423.1 (346.6; 444.9)	388.7 (352.3; 453.4) *	362.4 (331.2; 399.3) *	−39.2 (−95.5; 6.1) *	**0.0569**
GLY	244.8 (218.4; 312.5)	263.3 (227.2; 325.3)	257.8 (224.4; 293.1)	12.9 (−46.4; 42.8)	0.5188	254.3 (210.3; 318.1)	261 (232.2; 319.4)	265.3 (225.7; 307.8)	4.3 (−25.6; 27.7)	0.3903
SER	131.5 (117.7; 145.9)	130.9 (119; 141.8)	135 (127.9; 141.6)	6.9 (−20.5; 23.4)	**0.0439**	130.7 (115.2; 148.3)	138.2 (125.6; 150.3)	136.6 (129.5; 153.5)	10.1 (−8.7; 20.9)	0.0754
PRO	174.5 (154.4; 207.9)	159.1 (142.2; 175.7)	149.9 (142.6; 162.6)	−31.9 (−52.1; 3.4)	**0.0027**	177.2 (160; 199.1)	165.2 (151.3; 188.7)	155.9 (147.5; 168.8)	−20.5 (−38.7; −3.7)	**0.0008**
GLU	88.2 (81; 101.7)	79.1 (74.2; 82.1)	83.3 (77.7; 88.9)	−2 (−13.8; 1.6)	**0.0011**	86.7 (80.5; 97.1)	78.6 (76.1; 85.2)	83.6 (81.1; 90.3)	−1.6 (−11.6; 6.6)	**0.0003**
TYR	82.6 (70.2; 97.6)	68.9 (61.6; 70.6)	71.3 (68.1; 75.4)	−12.8 (−29.1; 5.1)	**0.0004**	74.2 (65.5; 91.9)	69 (63.6; 74.1)	73.6 (69.1; 76.4)	−3 (−18.5; 9)	**0.0166**
ASP	50.2 (49.8; 50.9)	49.4 (48.9; 49.8)	49.5 (49; 50.6)	−0.6 (−1.5; 0.2)	**0.0002**	49.5 (48.9; 50.9) *	49.1 (48.7; 49.5)	49.6 (49.1; 50.2)	0.1 (−0.9; 0.7) *	0.1663
ASN	230.3 (206.5; 253)	219.3 (202; 238.8)	232.5 (199.6; 264.7)	0.6 (−31.3; 61.9)	0.6661	219.6 (205.4; 263.7)	237.3 (212.1; 268.7)	261.3 (233.8; 283.3) *	25.8 (0.1; 57.3)	**0.0046**
Essential amino acids										
VAL	243.3 (216.3; 264.9)	285.4 (252.1; 318.6)	275.4 (248.6; 327.5)	41.7 (−20.6; 93.2)	**0.0157**	237.1 (214; 257.1)	222.7 (204.4; 239.4) *	224.5 (214.5; 249.8) *	−0.1 (−24; 13.2) *	0.4389
LEU	148.3 (130.5; 158.8)	156.8 (142.5; 172.8)	164.2 (143.3; 178.2)	16.8 (−9.2; 33.9)	**0.0342**	140.3 (129.7; 153.1)	136.5 (130.3; 152.6) *	136.7 (129.1; 153.2) *	0 (−3.7; 7.1)	0.8890
ILE	88.2 (83.2; 92.7)	94.3 (86.1; 106.2)	95.4 (86.2; 111.6)	6.3 (−4.2; 21.1)	0.0960	87.5 (81.2; 94.9)	84.7 (80.2; 91.2) *	87.2 (80.8; 93.6) *	0.3 (−3.4; 5.7)	0.9926
THR	145.4 (118.6; 162.3)	126.7 (118.9; 139.4)	131.2 (113.8; 142.6)	−22.8 (−40.8; 25.2)	0.5188	139.5 (125.6; 164.6)	147.2 (137.3; 157.7) *	144.6 (133.1; 159) *	2.9 (−13.5; 22.4)	0.6956
MET	58.4 (55.9; 62.5)	56.3 (54.4; 57.6)	56.4 (54.8; 57.7)	−1.9 (−6; 0.1)	**0.0089**	57.7 (56.6; 60.1)	57.9 (56.9; 60.2) *	57.2 (56.4; 59.3) *	−0.9 (−2.4; 0.7)	0.2004
PHE	87.7 (81.9; 91.5)	82.6 (78; 89.3)	82.8 (80.2; 86.4)	−4.1 (−12.3; 0.4)	**0.0342**	85.9 (80; 91.9)	84.5 (80.3; 87.6)	83.1 (80.1; 89.4)	−1.9 (−8.2; 5.9)	0.1568
LYS	123.7 (84.5; 218.3)	118 (81.2; 128.3)	136.6 (125.7; 144.4)	2 (−79.8; 56.8)	**0.0312**	118.8 (81.7; 187.7)	118.7 (104.2; 137.6)	142.4 (133.2; 158) *	22.6 (−22.8; 63.7)	**0.0011**
TRP	147 (102.4; 337.4)	156.9 (140.9; 176.3)	143.4 (114.4; 156.4)	1.7 (−217.4; 44.8)	0.5523	137.8 (99.5; 323.6)	187.5 (163.7; 203.1) *	152.3 (130.6; 176.1)	3 (−142.9; 57.4)	**0.0002**
Other amino acids and derivatives										
ORN	510.4 (387.4; 712.8)	477.6 (366.6; 545.3)	561.2 (481.3; 609.8)	51.8 (−196.8; 216.1)	**0.0400**	481 (333.3; 716.3)	513.6 (411.9; 572.2)	597 (547.2; 673.2) *	91.9 (−69.8; 279.2)	**0.0011**
ABA	54.7 (50.9; 61.6)	71.8 (57.9; 82.9)	72.7 (61.4; 83)	15 (2.1; 26.3)	**0.0048**	55.7 (50.8; 60.4)	58.4 (54.7; 65.9) *	64.7 (58.3; 73.6) *	8 (−2; 20.7)	**0.0077**
HYP	118.9 (111.6; 129.7)	122 (111.9; 132.4)	114.9 (108.3; 131.5)	−0.9 (−18.1; 18.5)	0.6065	116.9 (112.9; 139.5)	116.2 (110.4; 121.9)	117.1 (113; 128.3)	−2.1 (−14.8; 15.8)	0.6246

(^1^)—Comparison between all study points with Friedman test. (*)—significant difference based on Mann–Whitney U test between KETO and STD groups at each time point and Δ T2–T0. The significant *p*-values are marked in bold.

**Table 2:** Urinary excretion of free amino acids and derivatives in KETO and STD groups before and at the end of the nutritional intervention. Data are given as the median (Q25; Q75) in nmol/g.

	KETO Group				STD Group			
	T0	T2	Δ T2–T0	*p*-Value ^1^	T0	T2	Δ T2–T0	*p*-Value ^1^
Non-essential amino acids								
ALA	187.2 (108.3; 231.8)	78 (32.4; 127.7)	−79.5 (−119.5; −57.3)	**0.0005**	164.5 (114.3; 241.4)	75.9 (50.7; 134.2)	−77.6 (−109.8; −42.7)	**0.0001**
GLY	894.7 (450.1; 1580.1)	321.9 (199.7; 517)	−464.1 (−1234.8; −213.6)	**<0.0001**	833.7 (627.5; 1463.9)	215.7 (131.2; 566.3)	−578.2 (−1123.1; −263.9)	**<0.0001**
SER	120.5 (82.6; 241.7)	79.2 (48.8; 127.2)	−44.5 (−106.6; −10.9)	**0.0002**	145.6 (107.4; 218.3)	60.5 (48.1; 97)	−80.9 (−140.5; −36.5)	**<0.0001**
PRO	8.2 (5.8; 12.4)	13.5 (6.1; 15.1)	2.9 (0.0; 7.0)	**0.0163**	6.2 (5.1; 9.3)	12.4 (9.9; 16.9)	5.9 (1.6; 8.8)	**<0.0001**
GLU	36.5 (28.2; 40.0)	29 (26.3; 38.1)	−2.9 (−12.8; 2.3)	**0.0198**	35.2 (30.6; 43.2)	31.9 (24.1; 36.2)	−6.5 (−14; 1.0)	**0.0017**
CYS	118.2 (82.6; 162.4)	84.1 (59.7; 144.8)	−30.7 (−55.3; 20.8)	0.0577	88 (63.5; 139.9)	97.5 (77.9; 128.8)	−9.3 (−46.9; 37.7)	0.7910
GLN	321.3 (233.1; 583.4)	342.9 (248.3; 1572)	−2.5 (−171.9; 1299.9)	0.4711	340.1 (260.2; 604.9)	320.9 (126.6; 799.3)	−50.2 (−295.9; 418.1)	0.9251
TYR	84 (46.5; 133.3)	54.6 (27.6; 110)	−18.3 (−39.3; 9.1)	**0.0345**	53.2 (36.9; 95.8)	58.3 (25.4; 143.7)	5.9 (−25.9; 32.1) *	0.4675
Essential amino acids								
VAL	57.9 (43.2; 67.4)	57.5 (36.5; 70.7)	2.4 (−21.2; 8.9)	0.4004	49.6 (46.3; 79.3)	44.6 (27.3; 63.9)	−18.3 (−29.6; −0.3) *	**0.0011**
LEU	24.3 (18.3; 41.6)	29 (19.9; 36.1)	1.1 (−8.4; 9.0)	0.8476	27.5 (18.9; 50)	20.5 (11.8; 36.3)	−11.5 (−19.5; −3.4) *	**0.0020**
ILE	14.8 (12.7; 19.2)	16.8 (12; 22.9)	2.8 (−2.5; 5.8)	0.2297	14.8 (11.7; 18.4)	15.5 (9.5; 20.8)	−2.4 (−5.8; 3.8)	0.3786
THR	51.5 (30.1; 86.7)	18.6 (11; 35.7)	−23.1 (−58; −13.7)	**<0.0001**	53.7 (29.1; 88.6)	19.7 (10.8; 32.2)	−33.7 (−75.2; −12.5)	**<0.0001**
MET	25.8 (14.9; 69.3)	12.2 (8.2; 19.8)	−13.1 (−46.2; −6.7)	**<0.0001**	33.2 (20.8; 44.9)	13.1 (9.2; 22.6)	−16.2 (−26.3; −5)	**<0.0001**
PHE	45.4 (37.3; 61.4)	63.3 (44.8; 87.8)	8.0 (−3.8; 47.0)	**0.0109**	44 (38.6; 58.2)	56.2 (38.9; 94.6)	8.1 (−1.3; 34)	**0.0042**
LYS	128.7 (70.8; 174.5)	108.6 (58.8; 159.6)	−6.2 (−75.4; 18.7)	0.2691	99.8 (73.6; 220.7)	84.8 (43.8; 164)	−32.9 (−71.6; 14.0)	**0.0129**
HIS	620.9 (426; 1157.3)	582 (471.5; 1001.1)	81 (−150.6; 168.8)	0.3871	476.2 (380.6; 1053.8)	554.2 (349.5; 1130)	15.9 (−265.4; 203.7)	0.9115
TRP	54.9 (35.4; 89.8)	55 (26.1; 94.4)	6.2 (−24.2; 18.9)	0.9617	43.2 (22.6; 78.8)	52 (28.2; 115.8)	9.6 (−1.6; 26.7)	**0.0196**
Other amino acids and derivatives								
BAIB	81.5 (53.9; 112.6)	209.8 (129; 383.6)	122.4 (16.4; 268.8)	**0.0001**	89.3 (61.9; 153)	82.7 (50; 153.9) *	−1.6 (−31.5; 23.0) *	0.7260
AAA	46.6 (31.9; 64.4)	31.4 (20.2; 40.8)	−15.8 (−27.8; −6.8)	**0.0002**	51.7 (34.5; 63.3)	30.3 (22.2; 41.7)	−19.5 (−37.2; −3.4)	**0.0027**
C-C	61 (30.5; 76.2)	32.7 (13.8; 48.9)	−21 (−41.6; −13.7)	**0.0004**	47.2 (35.2; 99.9)	28.5 (13.6; 52.3)	−26.2 (−59.7; −7.9)	**0.0006**

(^1^)—Comparison between T0 and T2 with Wilcoxon test. (*)—significant difference based on Mann–Whitney U between KETO and STD group at each time point and Δ T2–T0. The significant *p*-values are marked in bold.

## Data Availability

The original data presented in the study are openly available in RepOD at https://doi.org/10.18150/BYT0PA.
